# Galactose Enhances Oxidative Metabolism and Reveals Mitochondrial Dysfunction in Human Primary Muscle Cells

**DOI:** 10.1371/journal.pone.0028536

**Published:** 2011-12-15

**Authors:** Céline Aguer, Daniela Gambarotta, Ryan J. Mailloux, Cynthia Moffat, Robert Dent, Ruth McPherson, Mary-Ellen Harper

**Affiliations:** 1 Department of Biochemistry, Microbiology and Immunology, Faculty of Medicine, University of Ottawa, Ontario, Canada; 2 Ottawa Hospital Weight Management Clinic, Ottawa, Ontario, Canada; 3 Division of Cardiology, University of Ottawa Heart Institute, Ottawa, Ontario, Canada; University of Cordoba, Spain

## Abstract

**Background:**

Human primary myotubes are highly glycolytic when cultured in high glucose medium rendering it difficult to study mitochondrial dysfunction. Galactose is known to enhance mitochondrial metabolism and could be an excellent model to study mitochondrial dysfunction in human primary myotubes. The aim of the present study was to 1) characterize the effect of differentiating healthy human myoblasts in galactose on oxidative metabolism and 2) determine whether galactose can pinpoint a mitochondrial malfunction in post-diabetic myotubes.

**Methodology/Principal Findings:**

Oxygen consumption rate (OCR), lactate levels, mitochondrial content, citrate synthase and cytochrome C oxidase activities, and AMPK phosphorylation were determined in healthy myotubes differentiated in different sources/concentrations of carbohydrates: 25 mM glucose (high glucose (HG)), 5 mM glucose (low glucose (LG)) or 10 mM galactose (GAL). Effect of carbohydrates on OCR was also determined in myotubes derived from post-diabetic patients and matched obese non-diabetic subjects. OCR was significantly increased whereas anaerobic glycolysis was significantly decreased in GAL myotubes compared to LG or HG myotubes. This increased OCR in GAL myotubes occurred in conjunction with increased cytochrome C oxidase activity and expression, as well as increased AMPK phosphorylation. OCR of post-diabetic myotubes was not different than that of obese non-diabetic myotubes when differentiated in LG or HG. However, whereas GAL increased OCR in obese non-diabetic myotubes, it did not affect OCR in post-diabetic myotubes, leading to a significant difference in OCR between groups. The lack of an increase in OCR in post-diabetic myotubes differentiated in GAL was in relation with unaltered cytochrome C oxidase activity levels or AMPK phosphorylation.

**Conclusions/Significance:**

Our results indicate that differentiating human primary myoblasts in GAL enhances aerobic metabolism. Because this cell culture model elicited an abnormal response in cells from post-diabetic patients, it may be useful in further studies of the molecular mechanisms of mitochondrial dysfunction.

## Introduction

Human primary muscle cells are a widely used model system to study muscle metabolism and its related disorders. Cell culture of human primary myotubes offers not only an excellent and dynamic model for studying metabolism under standardized conditions, but provides an excellent system for studying metabolic disorders. Indeed, a number of studies have shown that human primary muscle cells retain the same metabolic phenotype as those previously evidenced *in vivo* in muscle [1], [2], [3], [4], [5], [6], [7], [8], [9], [10], [11]. The oxidative capacity of skeletal muscle is highly influenced by various genetic and environmental factors; cultured myotubes offer a unique model that separates these two influences on metabolic phenotype [7].

Although a valuable model system, cultured muscle cells are highly glycolytic when grown and differentiated under high glucose conditions relative to muscle tissue *in vivo* [12], complicating the study of mitochondrial dysfunction. Indeed, myoblasts are routinely cultured in a standard high glucose medium which can diminish mitochondrial function. In the same way, rapidly proliferating cancer cells are known to be highly glycolytic when grown in high glucose conditions [13], [14], [15], a phenomenon known as the Crabtree effect, when glucose inhibits oxidative phosphorylation [16]. This phenomenon is not restricted to cancer cells. Other types of cells including embryonic tissues [17] and proliferative thymocytes [18] show diminished oxidative metabolism when grown under high glucose conditions.

Whereas the production of pyruvate via glycolytic metabolism of glucose yields 2 net ATP, the production of pyruvate via glycolytic metabolism of galactose yields no net ATP, forcing cells to have an increased reliance on oxidative phosphorylation (OXPHOS) for energy [13], [19], [20], [21]. Indeed, different types of cells (e.g., cancer cells, primary fibroblasts) grown in a medium in which glucose is replaced with galactose show a significantly increased oxygen consumption rate compared to cells grown in medium containing a high concentration of glucose (25 mM) [13], [14], [15]. The more aerobic state of cells grown in galactose is mainly due to a decrease in ATP production via anaerobic glycolysis [22] but is also thought to be due to a modulation of mitochondrial structure [15] and an increase in mitochondrial oxidative capacity as evidenced by increased OXPHOS protein expression and mitochondrial enzymatic activities [15]. Since cells grown in galactose rely mostly on oxidative phosphorylation to produce their ATP, they become more sensitive to mitochondrial toxins than cells grown in high glucose medium [13], [23], [24]. Another study has shown that primary skin fibroblasts isolated from patients with mitochondrial deficiency (*e.g.*, cytochrome oxidase deficiency, complex I deficiency, pyruvate dehydrogenase complex deficiency or with multiple respiratory chain defects) were not able to survive when cultured in a galactose-based medium [20]. Thus, culturing cells in a galactose medium seems to be a good alternative to high glucose medium in order to study mitochondrial dysfunction.

Obesity-associated insulin resistance and type 2 diabetes mellitus (T2DM) is characterized by a decrease in mitochondrial function. Mitochondrial respiration is known to be reduced in the skeletal muscle of T2DM patients [25], [26], [27] and lean insulin-resistant offspring of T2DM parents [28], [29]. Some studies, done on primary muscle cells derived from diabetic patients and matched controls have also shown a decrease in mitochondrial capacity: impaired ATP synthesis [30], [31], perturbed palmitate beta-oxidation when exposed to high level of palmitate [9], and impaired citrate synthase activity [7]. However, to our knowledge, no study has directly assessed whether myotubes derived from T2DM patients or patients with a history of T2DM retain a decrease in mitochondrial respiration when cultured *in vitro*. As mentioned above, this is most likely due to the fact that *in vitro*, primary human myotubes are mainly glycolytic rendering difficult the study of mitochondrial dysfunction in this model. The use of galactose medium instead of glucose medium to induce a metabolic shift towards a more oxidative phenotype could be a good model to study mitochondrial dysfunction in relation to T2DM and insulin resistance. However, to date, the response of human primary muscle cells to galactose has never been assessed. Here, we characterized the effect of replacing glucose with galactose on aerobic metabolism in primary muscle cells derived from lean and healthy donors. We have shown that differentiating primary human muscle cells in a galactose-replete medium enhances aerobic metabolism without altering mitochondrial content. Moreover, we have shown that this model system can be employed to investigate the mitochondrial bioenergetics of human myotubes derived from post-diabetic patients. To our knowledge, this is the first study that tests the impact of different sources of carbohydrates on human myotube bioenergetics. The value of this model system and its importance to investigating mitochondrial dysfunction in cell culture is also discussed herein.

## Results

### Impact of different carbohydrates on cellular physiology and ATP content

**Figure 1:**
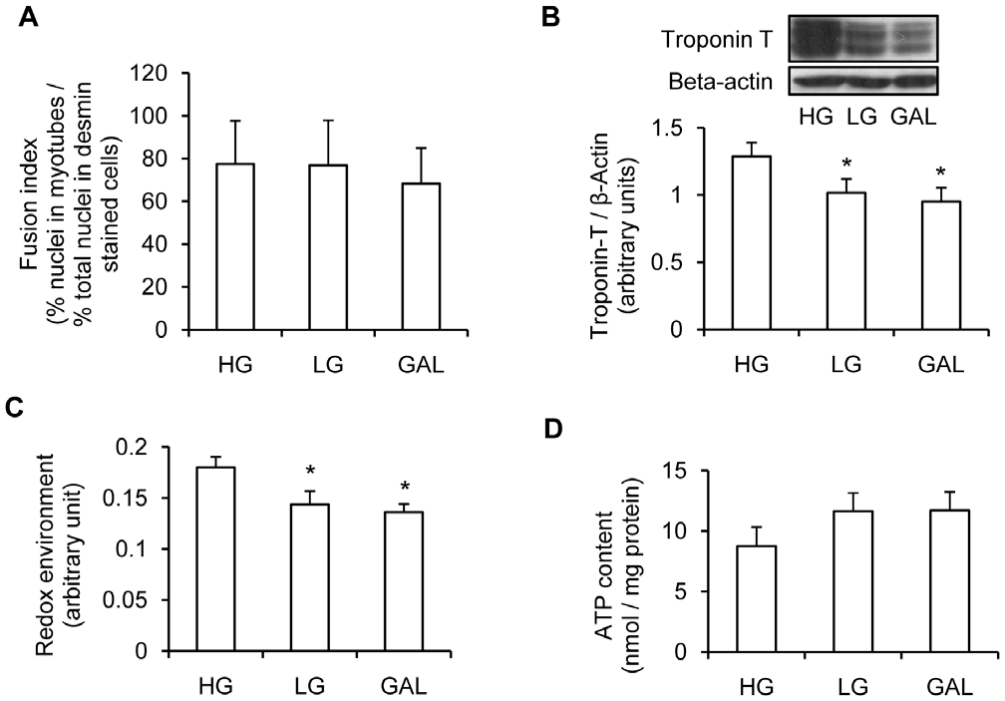
Effect of replacing a glucose medium with a galactose medium on myotube differentiation, redox environment and ATP content. **A.** Fusion index was measured in myotubes differentiated in HG (25 mM glucose), LG (5 mM glucose) or GAL (10 mM galactose) media for 7 days. The fusion index has been measured as the number of nuclei in differentiated myotubes (>2 myonuclei) as the percentage of the total number of nuclei in muscle cells (determined by a desmin immunofluorescence staining). Data are shown as mean ± SEM, n = 3. **B.** Myotube differentiation was assessed by Troponin T expression after 7 days of differentiation in HG (25 mM glucose), LG (5 mM glucose) or GAL (10 mM galactose) media. Top panel: representative Western blot of Troponin T expression in myotubes differentiated for 7 days in HG (25 mM glucose), LG (5 mM glucose) or GAL (10 mM galactose). Beta-actin was used as a loading control. Bottom panel: quantification by densitometry of Troponin T expression. Results are normalized to beta-actin expression. Data are shown as mean ± SEM, n = 4. *, p<0.05, LG and GAL vs HG. **C.** Myotube redox environment in response to differentiation in HG (25 mM glucose), LG (5 mM glucose) or GAL (10 mM galactose) was assessed using the MTT assay as described in the Methods section. Data are presented as mean ±SEM, n = 3, in which each condition was assessed in 6 replicates. **D.** ATP content in myotubes differentiated for 7 days in HG (25 mM glucose), LG (5 mM glucose) or GAL (10 mM galactose). Results are presented as means ± SEM, n = 4, in which each condition was assessed in duplicate.

Glucose restriction has been shown to decrease myotube differentiation [32]. The fusion index in cells differentiated in high glucose (25 mM, HG), low glucose (5 mM, LG) or GAL was not significantly different between the 3 differentiation conditions (Fig. 1A). However, troponin T expression, which is another marker of myotube differentiation, was significantly decreased in cells differentiated in LG or GAL compared to cells differentiated in HG (p<0.05, Fig. 1B). Yet, cells differentiated in GAL showed the same level of differentiation than cells differentiating in LG.

After 7 days of differentiation in the different mediums, the redox state of the cells was assessed using the MTT (3-(4,5-dimethylthiazol-2-yl)-2,5-diphenyl tetrasodium bromide) assay. A significant decrease in the redox state was found in myotubes differentiated in LG or GAL compared to HG (p<0.05), with no difference between cells differentiated in LG and GAL (Fig. 1C).

We also measured ATP content in myotubes differentiated for 7 days in HG, LG or GAL. As shown in Figure 1D, no significant difference was found in ATP content between myotubes differentiated in the three types of media. Hence, these observations indicate that replacing glucose with galactose does not have a negative effect on cellular physiology and metabolism.

### Replacing glucose with galactose in differentiation medium increases myotube aerobic capacity

**Figure 2:**
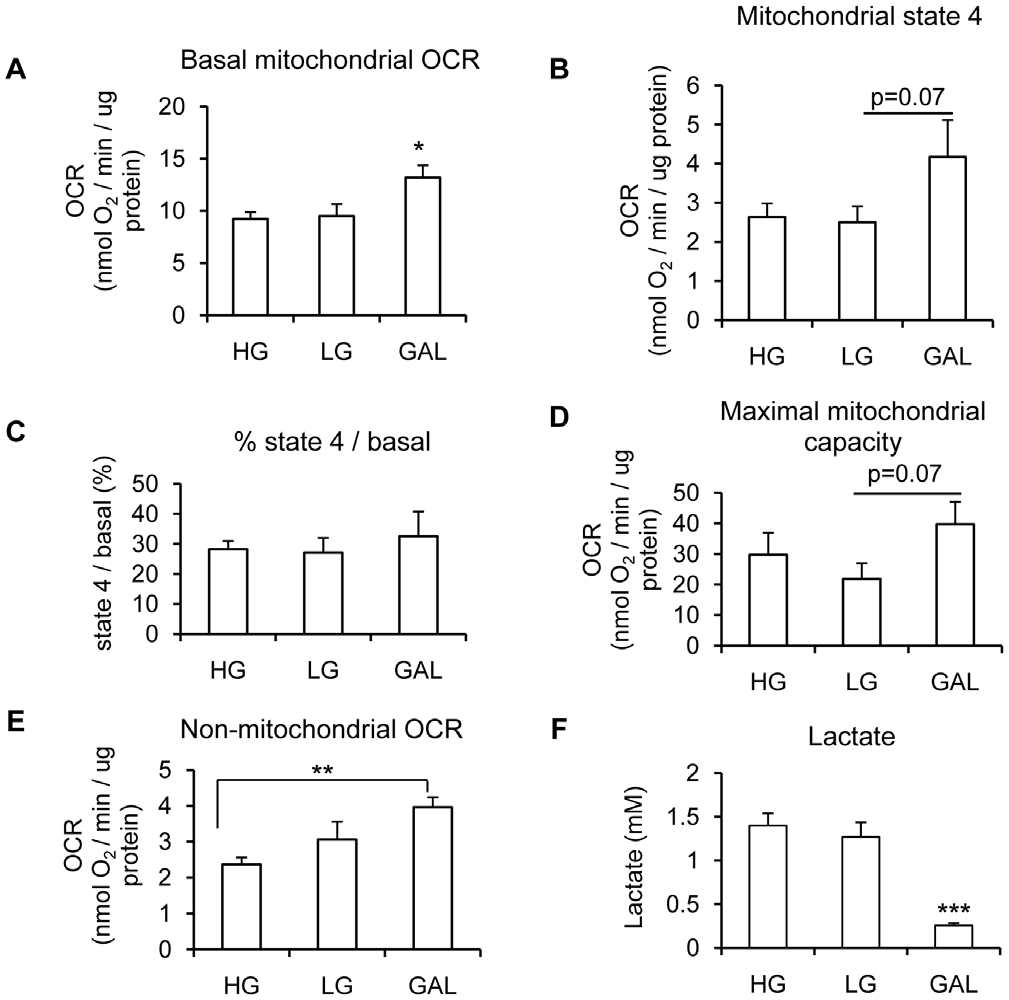
Effect of replacing a glucose medium with a galactose medium on myotube aerobic capacity. **A.** Basal mitochondrial oxygen consumption rate. *, p<0.05, GAL vs HG and LG. **B.** State 4 respiration (leak-dependent; non-phosphorylating). After basal oxygen consumption rate measurement, cells were treated with oligomycin (600 ng/ml) to determine state 4 respiration. p = 0.06, GAL vs LG. **C.** Percentage of basal OCR due to proton leak was calculated from the data shown in Figure 2A and B. Data are presented as means ± SEM, n = 7, in which each condition was assessed in 5–6 replicates. **D.** Maximal mitochondrial oxygen consumption capacity. After basal and state 4 respiration measurements, cells were treated with FCCP (1 µM) to determine maximal oxygen consumption. *, p<0.05, GAL vs LG. **E.** Non-mitochondrial oxygen consumption rate. After basal, state and maximal respiration measurements, cells were treated with antimycin (4 µM) to determine non-mitochondrial oxygen consumption. **, p<0.01, GAL vs LG. **F.** Lactate concentration in the extracellular media of myotubes differentiated for 7 days in HG (25 mM glucose), LG (5 mM glucose) or GAL (10 mM galactose). Results are presented as means ± SEM, n = 7, in which each condition was assessed in duplicate. ***, p<0.0001, GAL vs HG and LG.

Primary human muscle cells differentiated in GAL showed a ∼40% increase in basal mitochondrial oxygen consumption rate (basal mitochondrial OCR = basal OCR – non-mitochondrial OCR) compared to cells differentiated in either HG or LG (p<0.05, Fig. 2A). Mitochondrial state 4 respiration (proton leak dependent respiration = (OCR after oligomycin treatment – non-mitochondrial OCR)) tended to be higher in GAL myotubes compared to LG myotubes (p = 0.07) or HG myotubes (p = 0.1) (Fig. 2B) without reaching significance. However, the percentage of basal mitochondrial OCR due to proton leak was not significantly different between treatments (Fig. 2C). There was also a trend for a higher maximal mitochondrial OCR (measured after FCCP treatment) in GAL cells compared to LG cells, but this did not reach significance (p = 0.07, Fig. 2D).

The non-mitochondrial OCR was measured by treating cells with antimycin A. Intriguingly, OCR after antimycin A treatment was significantly higher in myotubes differentiated in GAL compared to myotubes differentiated in HG (p<0.01, Fig. 2E).

To test the metabolic changes accompanying differentiating cells in GAL further, we measured the amount of lactate released in the medium over a 24 h period. As shown in Figure 2F, cells differentiated in GAL showed a significant decrease in lactate production compared to cells differentiated in LG or HG indicating a decrease in anaerobic metabolism in cells differentiated in GAL (p<0.0001). No significant difference in lactate production was found between myotubes differentiated in HG and LG.

Taken together, these data confirm that myotubes differentiated in presence of glucose are mostly glycolytic, and that the replacement of glucose by galactose in the differentiation medium forces cells to adopt a more oxidative metabolic state.

### Effect of galactose on mitochondrial content and enzyme activity

**Figure 3:**
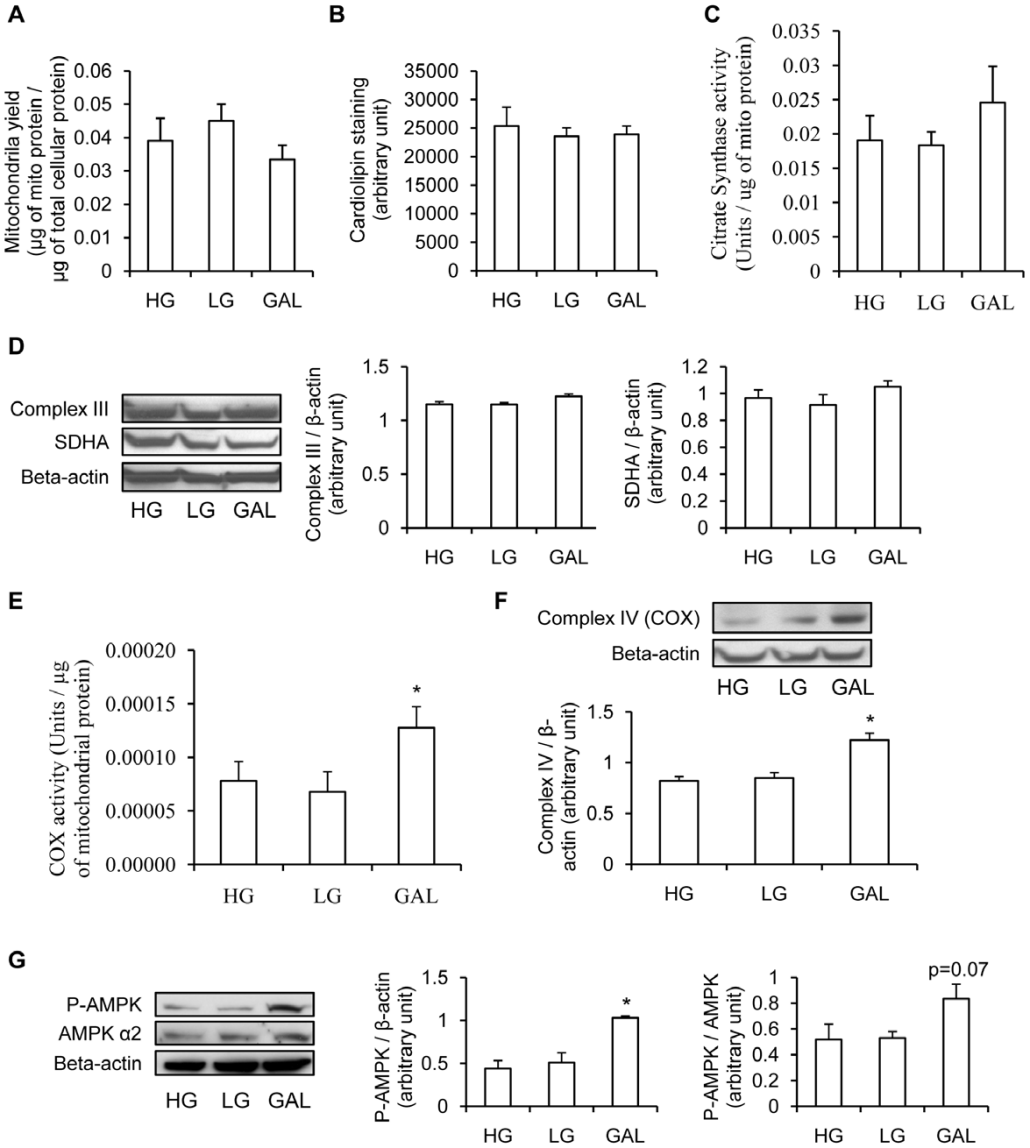
Effect of replacing a glucose medium with a galactose medium on mitochondrial markers. **A.** Mitochondrial yield measured in myotubes differentiated for 7 days in HG (25 mM glucose), LG (5 mM glucose) or GAL (10 mM galactose). Mitochondrial yield was determined as mitochondrial protein content per total cellular protein content. Results are presented as means ± SEM, n = 6, in which each condition was assessed in duplicate. **B.** Cardiolipin staining in myotubes differentiated for 7 days in HG (25 mM glucose), LG (5 mM glucose) or GAL (10 mM galactose). Cardiolipin was stained with 10N-nonyl acridine orange (NAO, 1 µM) in fixed myotubes for 30 min. The intensity of the staining was then measured by fluorescence. Results are presented as means ± SEM, n = 3. **C.** Citrate synthase activity measured on isolated mitochondria from myotubes differentiated for 7 days in HG (25 mM glucose), LG (5 mM glucose) or GAL (10 mM galactose). Results are presented as means ± SEM, n = 5, in which each condition was assessed in duplicate. **D.** Left panel: representative Western blot of Complex III and SDHA expression in myotubes differentiated for 7 days in HG (25 mM glucose), LG (5 mM glucose) or GAL (10 mM galactose). Complex III and SDHA were used as a mitochondrial content marker and beta-actin as a loading control. Right panels: quantification by densitometry of complex III and SDHA expressions. Data are presented normalized to beta-actin expression. Data are shown as mean ±SEM, n = 4. **E.** Cytochrome C oxidase activity measured in isolated mitochondria from myotubes differentiated for 7 days in HG (25 mM glucose), LG (5 mM glucose) or GAL (10 mM galactose). Results are presented as means ± SEM, n = 5, in which each condition was assessed in duplicate. *, p<0.05, GAL vs HG and LG. **F.** Top panel: representative Western blot of Complex IV expression in myotubes differentiated for 7 days in HG (25 mM glucose), LG (5 mM glucose) or GAL (10 mM galactose). Beta-actin was used as a loading control. Bottom panel: quantification by densitometry of complex IV expression. Data are presented normalized to beta-actin expression. Data are shown as mean ±SEM, n = 4. *, p<0.05, GAL vs HG and LG. **G.** Left panel: representative Western blot of P-AMPK expression in myotubes differentiated for 7 days in HG (25 mM glucose), LG (5 mM glucose) or GAL (10 mM galactose). Beta-actin and AMPKα2 were used as loading controls. Right panels: quantification by densitometry of P-AMPK. Data are presented normalized to beta-actin and AMPKα2 expression. Data are shown as mean ±SEM, n = 3. *, p<0.05, GAL vs HG and LG.

In order to determine if the increase in OCR shown in Figure 2 was the result of an increase in mitochondrial biogenesis, different mitochondrial markers were measured. First, mitochondrial yield was determined as mitochondria protein content per total cellular protein content. As shown in Figure 3A, differentiating the cells in GAL did not have any effect on mitochondrial yield compared to myotubes differentiating in HG or LG. Another method to determine mitochondrial content is the staining of cardiolipin with 10N-nonyl acridine orange (NAO). As shown on Figure 3B, this method corroborates that differentiating the cells in HG, LG or GAL do not affect mitochondrial content. These results were confirmed by measuring the protein levels of the mitochondrial markers, complex III and SDHA. The levels of each protein were not significantly affected by the different carbohydrate sources (Fig. 3D).

The impact of differentiating cells in galactose on the activity of citrate synthase and cytochrome C oxidase (COX) was also examined. The activities were measured on isolated mitochondria to determine whether the capacity of the TCA (tricarboxylic acid) cycle or the electron transport chain was increased in GAL myotubes, respectively. Citrate synthase activity was not changed by GAL medium compared to either HG or LG medium (Fig. 3C). However, COX activity was significantly higher in GAL myotubes compared to both HG and LG myotubes (p<0.01, Fig. 3E). This higher COX activity was in relation with a higher COX expression level in myotubes differentiating in GAL compared to LG or HG (p<0.05, Fig. 3F).

AMPK is a major metabolic sensor that is activated by an increase in the ratio of AMP/ATP in order to restore energy status of the cell trough the stimulation of ATP-producing processes (*e.g.,* glucose uptake, fatty acid oxidation, and mitochondrial biogenesis) [33], [34] and the inhibition of ATP-consuming processes (fatty acid synthesis, glycogen synthesis, and protein synthesis) [35], [36], [37]. To determine if the differentiation of cells in GAL medium also affects AMPK activity, we estimated AMPK activity by measuring AMPK phosphorylation (P-AMPK) in cells differentiated in either HG, LG or GAL for 7 days. As shown on Figure 3G, P-AMPK was higher in cells differentiated in GAL compared to LG or HG (p<0.05 for P-AMPK/β-actin; p = 0.07 for P-AMPK/AMPK).

### Acute exposure to galactose does not affect myotube oxidative capacity

**Figure 4:**
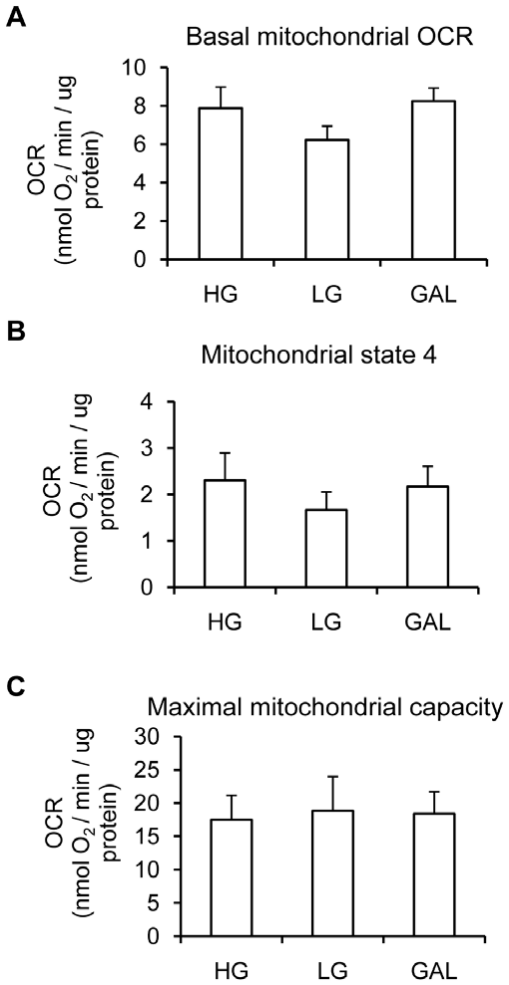
Effect of an acute treatment with galactose medium on myotube aerobic capacity. **A.** Basal oxygen consumption rate. **B.** State 4 respiration (leak dependent; non-phosphorylating). After basal oxygen consumption rate measurement, cells were treated with oligomycin (600 ng/ml) to determine state 4 respiration. **C.** Maximal oxygen consumption capacity. After basal and state 4 respiration, cells were treated with FCCP (1 µM) to determine maximal oxygen consumption. **A–C.** Myotubes were differentiated for 7 days in LG (5 mM glucose). On the 7^th^ day of differentiation, myotubes were exposed to HG (25 mM), LG (5 mM glucose) or GAL (10 mM galactose) for 45 min before measuring basal oxygen consumption rate. Results are presented as means ± SEM, n = 4, in which each condition was assessed in 5 replicates.

In order to determine if an acute treatment was sufficient to improve the aerobic capacity of the myotubes, cells were differentiated for 7 days in LG, and then incubated for 45 min before measuring oxygen consumption in HG, LG or GAL media. As shown in Figure 4, basal mitochondrial (Fig. 4A), mitochondrial state 4 (Fig. 4B), and maximal mitochondrial OCR (Fig. 4C) were unaffected by an acute treatment with GAL. Hence, these data indicate that in order to induce a metabolic shift towards increased oxidative metabolism, cells have to be exposed to GAL for a prolonged period.

### Post-diabetic myotubes show incapacity to increase their oxidative metabolism when differentiated in galactose medium

**Figure 5:**
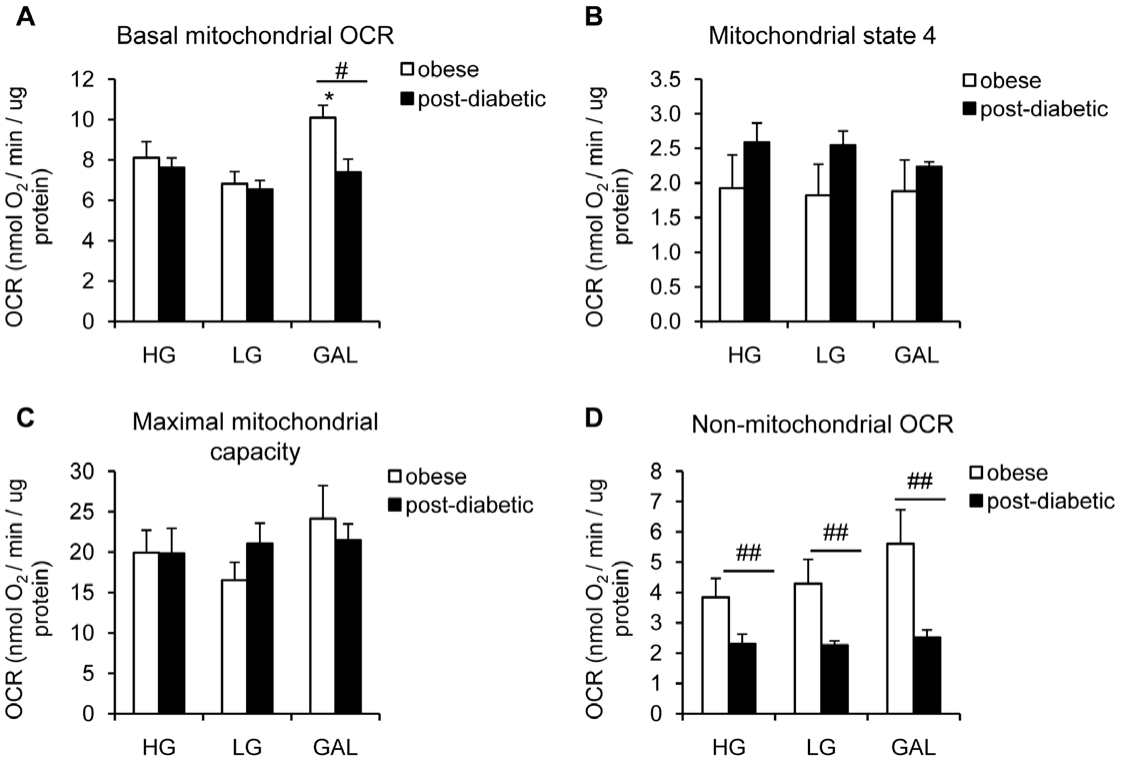
Absence of an increase in basal oxygen consumption in post-diabetic myotubes differentiated in galactose media. **A.** Basal oxygen consumption rate. *, p<0.05, GAL vs HG and LG. #, p<0.05, post-diabetic vs obese. **B.** State 4 respiration (leak dependent; non-phosphorylating). After basal oxygen consumption rate measurement, cells were treated with oligomycin (600 ng/ml) to determine state 4 respiration. **C.** Maximal oxygen consumption capacity. After basal and state 4 respiration, cells were treated with FCCP (1 µM) to determine maximal oxygen consumption. **D.** Non-mitochondrial oxygen consumption rate. After basal, state and maximal respiration, cells were treated with antimycin (4 µM) to determine non-mitochondrial oxygen consumption. ##, p<0.001, post-diabetic vs obese. **A–D.** Myotubes were differentiated for 7 days in HG (25 mM glucose), LG (5 mM glucose) or GAL (10 mM galactose). Results are presented as means ± SEM, n = 5, in which each condition was assessed in 5–6 replicates.

The observed effect of galactose on oxidative metabolism prompted us to test the effect of this carbohydrate on the aerobic function of muscle cells collected from patients with a history of diabetes (post-diabetic patients). To test if myotubes derived from obese post-diabetic patients were responsive to GAL at the level of OCR, post-diabetic myotubes and their matched obese non-diabetic myotubes were differentiated for 7 days in HG, LG or GAL. When differentiated in LG or HG, post-diabetic myotubes showed the same basal mitochondrial OCR as obese non-diabetic myotubes (Fig. 5A). However, unlike obese non-diabetic myotubes, basal mitochondrial OCR in post-diabetic myotubes showed no response to GAL, leading to a significant difference between groups (Fig. 5A; p<0.05). This interesting result highlights a defect in mitochondrial function in post-diabetic myotubes. Mitochondrial state 4 OCR (Fig. 5B) and maximal mitochondrial capacity (Fig. 5C) were however not differentially affected by GAL, or different between post-diabetic myotubes and obese non-diabetic myotubes. Interestingly, non-mitochondrial OCR (in the presence of saturating antimycin) was significantly lower in post-diabetic myotubes compared to obese non-diabetic myotubes in the 3 different conditions (Fig. 5D).

### Post-diabetic myotubes show no increases in COX activity or P-AMPK when differentiated in galactose medium compared to low or high glucose media

**Figure 6:**
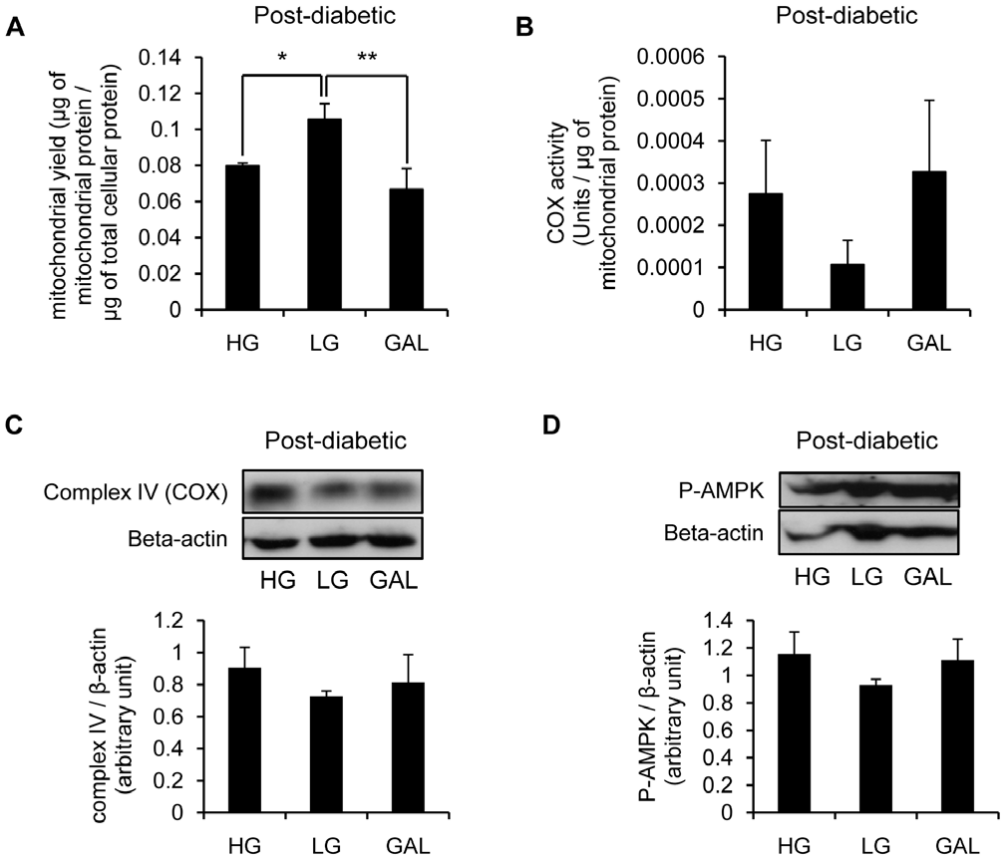
Absence of an increase in cytochrome C oxidase activity and AMPK phosphorylation in post-diabetic myotubes differentiated in galactose media. **A.** Mitochondrial yield measured in post-diabetic myotubes differentiated for 7 days in HG (25 mM glucose), LG (5 mM glucose) or GAL (10 mM galactose). Mitochondrial yield was determined as mitochondrial protein content per total cellular protein content. Results are presented as means ± SEM, n = 4. *, p<0.05, LG vs HG, **, p<0.01 LG vs GAL. **B.** Cytochrome C oxidase activity measured in isolated mitochondria from post-diabetic myotubes differentiated for 7 days in HG (25 mM glucose), LG (5 mM glucose) or GAL (10 mM galactose). Results are presented as means ± SEM, n = 4. *, p<0.05, GAL vs HG and LG. **C.** Top panel: representative Western blot of Complex IV expression in post-diabetic myotubes differentiated for 7 days in HG (25 mM glucose), LG (5 mM glucose) or GAL (10 mM galactose). Beta-actin was used as a loading control. Bottom panel: quantification by densitometry of complex IV expression. Data are presented normalized to beta-actin expression. Data are shown as mean ±SEM, n = 4. **D.** Left panel: representative Western blot of P-AMPK expression in post-diabetic myotubes differentiated for 7 days in HG (25 mM glucose), LG (5 mM glucose) or GAL (10 mM galactose). Beta-actin was used as loading controls. Right panels: quantification by densitometry of P-AMPK. Data are presented normalized to beta-actin and AMPKα2 expression. Data are shown as mean ±SEM, n = 4.

To identify why post-diabetic myotubes are incapable of increasing oxidative metabolism in response to GAL, we measured mitochondrial content, and COX expression and activity (Fig. 6). Surprisingly, we found a significant increased mitochondrial yield in post-diabetic myotubes differentiated in LG compared with myotubes differentiated in both HG (p = 0.05) and GAL (p<0.01) (Fig. 6A). However, COX activity was not significantly different between conditions due to the high variability in activity between post-diabetic samples (Fig. 6B). Furthermore, COX expression was not significantly increased when post-diabetic cells were differentiated in GAL compared to LG or HG (Fig. 6C). We also measured the level of P-AMPK in post-diabetic myotubes differentiated in HG, LG or GAL. In contrast to control myotubes (Figure 3G), post-diabetic myotubes did not show increased AMPK phosphorylation in response to GAL medium (Fig. 6D).

## Discussion

The present study identifies galactose differentiation medium as a useful experimental system to analyze mitochondrial metabolism in human primary muscle cells. Our findings demonstrate that this system provides an effective method to elucidate mitochondrial dysfunction in myotubes derived from post-diabetic patients. Our main results show that OCR was 30% higher in healthy myotubes differentiating in GAL compared to LG or HG media, which is due, at least in part, to an increase in AMPK phosphorylation and COX activity in GAL treated cells. When differentiated in LG or HG media, post-diabetic myotubes had similar OCR as myotubes derived from matched obese non-diabetic volunteers. Interestingly, unlike obese non-diabetic myotubes, post-diabetic myotubes were unable to increase their OCR in response to GAL medium. Post-diabetic myotubes were also unable to increase AMPK phosophorylation and COX activity when differentiated in GAL. To our knowledge, this study is the first to show that the use of a GAL medium is a simple means to increase OCR in human primary muscle cells and to potentially identify mitochondrial dysfunction.

To better characterize the effect of different carbohydrate sources and/or concentration during cell culture, we first determined the level of differentiation in myotubes differentiated for 7 days in LG (5 mM glucose), HG (25 mM glucose) or GAL (10 mM galactose). Myotubes differentiated in HG showed the highest level of differentiation as indicated by troponin T expression level, which is commonly used as a marker of muscle cell differentiation [8], [38]. Thus, we have confirmed in our model (human primary muscle cells) that glucose availability can affect the level of muscle cell differentiation as previously reported by others [32] in muscle cell lines (C2C12) and mouse primary muscle cells. However, there were no differences in the levels of differentiation in myotubes differentiated in 5 mM glucose (LG) compared to 10 mM galactose (GAL), showing that the metabolic differences found between LG and GAL myotubes were not the result of alteration in levels of differentiation.

As previously shown by others in different cell lines or primary fibroblasts [13], [14], [15], replacing glucose with galactose in the medium forces the cells to become more oxidative in order to maintain ATP levels. In accordance with an increased oxidative metabolism, differentiating the cells in GAL decreased anaerobic metabolism as shown by the diminished generation of lactate. The sharp decrease in lactate production by the myotubes differentiated in GAL is not surprising since the production of pyruvate through galactose metabolism yields no net ATP, forcing the cells to rely on aerobic metabolism instead of anaerobic glycolysis to produce ATP [13], [19], [20], [21]. The increase in oxygen consumption rate in cells differentiated in GAL was not due to an increase in proton leak-dependent oxygen consumption rate as shown by the proportion of total respiration that was due to proton leak. A trend for a higher maximal respiration rate in myotubes differentiated in GAL compared to LG was also found, but this did not reach significance (p = 0.07). The higher mitochondrial OCR in GAL cells was not the result of an increase in mitochondrial content as evidenced by the similar levels of various mitochondrial markers (mitochondrial yield, cardiolipin staining, SDHA and complex III expressions). Furthermore, citrate synthase activity, commonly used as a marker of TCA cycle activity, was not affected by the different sources of carbohydrates. Interestingly, this increase in mitochondrial OCR was related to a ∼70% increase in mitochondrial COX activity certainly due to an increase in COX expression. Our results on human primary muscle cells are in accordance with a previous report showing increases in COX expression and activity in HeLa cells grown in 10 mM galactose compared to 25 mM glucose [15]. We also found an increased AMPK phosphorylation in myotubes differentiated in GAL compared to LG or HG. AMPK is a major metabolic sensor in cells and is activated by increased AMP/ATP for the purpose of restoring the energy status of the cell [33], [34], [35], [36], [37]. An increase AMPK phosphorylation in response to a decrease in glucose availability (25 mM compared to 5 mM) has also been found by others in C2C12 myotubes [32]. In our primary muscle cell model, we were not able to detect an increased P-AMPK when cells were differentiated in 5 mM glucose compared to 25 mM glucose, consistent with the conclusion that 5 mM glucose is not restrictive enough in our model. However, a nice increase in P-AMPK was found when myotubes were differentiated in GAL, suggesting that GAL medium induced a deprivation of energy, which in turn did activate AMPK in order to restore energy status of the cells. Thus, the increased COX activity and oxygen consumption in GAL myotubes could be the result of an increased AMPK phosphorylation. Other studies will be needed to test this hypothesis.

To test if an acute treatment with GAL could have the same effect as differentiating the cells for 7 days in GAL, myotubes were differentiated for 7 days in LG medium and then acutely treated for 45 min with HG, LG or GAL before OCR measurement. This acute treatment with GAL was not sufficient to increase OCR compared to LG or HG treatments. This may be attributed to the fact that glycolytic intermediates would not be completely depleted after a 45 min incubation in GAL medium. Taken together, those results show that differentiating human primary myotubes in GAL for 7 days could be an easy way to improve the aerobic capacity of these cells and decrease their reliance on anaerobic glycolysis.

An unexpected and intriguing finding was the increase in non-mitochondrial OCR in myotubes differentiated in GAL compared to HG. Extra-mitochondrial sites for oxygen consumption include the nicotinamide adenine dinucleotide phosphate (NADPH) oxidase, nitric oxide synthase, and the xanthine oxidase. Measurement of the cell redox environment with the MTT assay is based on a reaction in which the formed NAD(P)H reduces tetrazolium (MTT) reagent to a blue formazan. The intensity of the reduced product colour is thus proportionate to the NAD(P)^+^/NAD(P)H concentration in the cells [39]. Since the redox environment measured with the MTT assay was significantly lower in LG and GAL cells compared to HG cells, this could indicate a decrease in reduced pyridine nucleotide levels in LG and GAL myotubes compared to HG myotubes. A similar decrease in redox state was also found by others in HeLA cells grown in 10 mM galactose compared to 25 mM glucose [15]. Further studies are needed to directly assess NAD(P)H level and NAD(P)H oxidase activity to confirm this hypothesis.

Galactose medium is often used to study the effect of mitochondrial toxins in cancer cells [13], [23], [24] and has also been used to examine mitochondrial dysfunction in primary skin fibroblasts derived from patients with defect in mitochondrial function [20]. In the present study, we have shown, not only that GAL is able to increase the oxidative metabolism of healthy human primary muscle cells, but also that GAL can be used to identify mitochondrial dysfunction in primary muscle cells derived from post-diabetic patients. Indeed, unlike myotubes derived from matched obese non-diabetic subjects, myotubes derived from post-diabetic patients were not able to increase their oxygen consumption rate when differentiated in GAL. Thus, in spite of the fact that there was no difference in OCR between obese non-diabetic myotubes and post-diabetic myotubes when differentiated in LG or HG, the GAL media was able to highlight a decreased oxidative capacity in post-diabetic myotubes compared to obese non-diabetic myotubes. Other studies have described a decrease in oxidative metabolic capacity in myotubes derived from diabetic patients as indicated by a decrease in citrate synthase activity [7] and a decrease in lipid oxidation capacity [5], [9]. Myotubes derived from post-diabetic subjects are also known to have a decreased oxidative capacity when challenged with a high glucose medium (decreased mitochondrial content, decreased citrate synthase and COX activities) compared to myotubes derived from matched obese non-diabetic individuals [40]. Despite this decrease oxidative capacity [5], [9], [40], no difference in OCR was found in the present study between obese non-diabetic and post-diabetic myotubes differentiated in either HG or LG. This is presumably due to the fact that under these conditions, myotubes were highly glycolytic rendering it more difficult to detect a decrease in OCR in post-diabetic compared to obese non-diabetic myotubes. However, the GAL medium was able to identify decreased OCR in post-diabetic myotubes, revealing the utility of this approach to detect mitochondrial dysfunction *in vitro*. Our results also confirm that post-diabetic myotubes display an abnormal metabolic flexibility when challenged with different substrates (here with galactose) as previously published by others who studied myotubes derived from (post-)diabetic, insulin resistant or obese patients when challenged with high fat levels [9], [11], [41] or high glucose levels [40]. In the present study, the lack of an increase in OCR in post-diabetic myotubes appears to be related to unaltered COX expression or activity levels or AMPK phosphorylation. Another study showed that myotubes derived from diabetic patients lack the capacity to activate the AMPK pathway (measured as acetyl-CoA carboxylase phosphorylation) in response to of palmitate, leading to an absence of any increase in myotubes palmitate oxidation [9]. It seems that myotubes derived from (post-)diabetic patients have a impaired AMPK activity in response to different AMPK-stimulating agents. Other studies will be needed to determine whether AMPK stimulation is truly impaired in (post-)diabetic muscle and to determine the mechanisms causing AMPK “dysfunction”.

Interestingly, we also found a significant decrease in non-mitochondrial OCR in post-diabetic myotubes compared to matched obese non-diabetic myotubes, independently of the source of carbohydrates used in the differentiation medium. This result is in accordance with a study from our group showing perturbations in NADPH production due to an impaired glucose-6-phosphate dehydrogenase in post-diabetic myotubes [42]. This could be due to a defect in the pentose phosphate pathway in post-diabetic myotubes. Another hypothesis explaining the lower non-mitochondrial OCR in post-diabetic myotubes compared to obese control myotubes is a decrease in NADPH oxidase protein content or activity. NADPH oxidase produces superoxide by coupling their electrons to oxygen. Thus this is an enzyme involved in reactive oxygen species production (ROS). Interestingly, the measurement of ROS (by the DCFH-DA assay) in post-diabetic and obese non-diabetic myotubes showed lower ROS content in post-diabetic myotubes (data not shown). Studying the mechanism underlying this phenomenon was not the purpose of our study and needs further investigation.

To our knowledge, this is the first study to test the impact of different carbohydrate sources on human myotube bioenergetics. Furthermore, this is the first study that has directly assessed oxygen consumption rate *in vitro* in myotubes derived from post-diabetic patients and demonstrated decrease oxygen consumption rates in post-diabetic myotubes compared to obese non-diabetic myotubes. Moreover, we have shown that differentiating cells in GAL is an excellent model system to investigate the mitochondrial bioenergetics of human myotubes derived from patients with a history of T2DM. The use of this model could enable further research to better understand the molecular mechanisms leading to mitochondrial dysfunction during the development of insulin resistance and other metabolic disorders in skeletal muscle.

## Materials and Methods

### Ethics statement

All participants gave informed consent and the experimental protocol was approved by the Human Research Ethics Committees of the Ottawa Hospital and the University of Ottawa Heart Institute.

### Muscle biopsy and primary human muscle cell culture

Biopsies of vastus lateralis were obtained from 7 lean and healthy participants (male/female: 6/1, age: 41 years ±6.7, BMI (Body Mass Index): 20.8 kg/m^2^ ±0.5) and from 5 obese non-diabetic persons (male/female: 4/1, age: 55 years ±2.1, BMI: 39.2 kg/m^2^ ±1.3) and 5 obese post-diabetic patients (male/female: 4/1, age: 54 years ±2.3, BMI: 36.6 kg/m^2^ ±1.3) after local anesthesia, using a 5 mm Bergstrom needle (Opitek, Glostrup, Denmark) as described previously by Costford *et al* [40]. The post-diabetic patients had a documented history of obesity-associated T2DM prior to a 26-week standard clinical weight loss program at the Ottawa Hospital Weight Management Clinic and were no longer diabetic after the weight loss program (blood glucose <6.1 mmol/l and HbA1c <5.7%). Characterization of primary muscle cells from the obese non-diabetic and the obese post-diabetic participants was previously published by our lab [40].

Subjects were fasted for 12 hours and refrained from physical activity for three days prior to the biopsy. 40 mg of the muscle biopsy were processed for satellite cell isolation and culture. The muscle biopsy was minced, subjected to a 30 minutes trypsin digestion and plated in Ham's F-10 medium supplemented with 15% Foetal Bovine Serum (FBS, Gibco, Burlington, ON, Canada), 0.5 mg/ml BSA, 1 µM dexamethazone (Invitrogen, Burlington, ON, Canada), 10 ng/ml Epidermal Growth Factor (EGF, Invitrogen, Burlington, ON, Canada) and 25 pM insulin (Sigma–Aldrich, Oakville, ON, Canada), and maintained in 37°C in humidified 95% air 5% CO_2_. Muscle satellite cells were isolated at ∼80% confluency using an immuno-based magnetic sorting technique as previously described [40]. Isolated primary muscle cells were grown in Ham's F-10 (6.11 mM glucose) (Gibco, Burlington, ON, Canada) supplemented with 12.5% FBS, 1 µM dexamethazone, 10 ng/ml EGF, 25 pM insulin, 1× antibiotic-antimycotic (Gibco, Burlington, ON, Canada) and 2.5 µg/ml gentamycin (Growth Medium, GM) to ∼90% confluency prior to differentiation in either low glucose DMEM (5.5 mM glucose, supplemented with 1 mM sodium pyruvate, 25 pM insulin, 2% horse serum, 1× antibiotic-antimycotic and 2.5 µg/ml gentamycin) (LG, Gibco, Burlington, ON, Canada), galactose DMEM (10 mM galactose (Sigma–Aldrich, Oakville, ON, Canada), 1 mM sodium pyruvate, supplemented with 25 pM insulin, 2% horse serum, 1% antibiotic-antimycotic and 2.5 µg/ml gentamycin) (GAL) or high glucose DMEM (25 mM glucose, supplemented with 1 mM sodium pyruvate, 25 pM insulin, 2% horse serum, 1% antibiotic-antimycotic and 2.5 µg/ml gentamycin) (HG) for 7 days prior to experimentation. The choice of 10 mM galactose was based on previous published studies on other cell lines [13], [14], [15]. For acute treatments (Figure 4), myotubes were differentiated in LG for 7 days, and then incubated for 45 min in HG, LG or GAL prior to experimentation.

### Immunofluorescence staining and fusion index

In order to calculate the fusion index, cells differentiated in HG, LG or GAL for 7 days were subjected to a desmin immunofluorescence staining as previously described [8]. The fusion index was determined as the number of nuclei in differentiated myotubes (desmin positive cells with more than 2 nuclei) as a percentage of the total number of nuclei in muscle cells (desmin positive cells) [43].

### Western Blots

Cell lysates were quantified for protein content and 30 µg of protein were separated by 8% SDS-polyacrylamide gel electrophoresis and then transferred to a nitrocellulose membrane. Following primary antibodies were used: monoclonal anti-troponin T (Sigma–Aldrich, Oakville, ON, Canada), anti-complex III (Invitrogen, Burlington, ON, Canada), anti-SDHA (succinate dehydrogenase, Santa Cruz Biotechnology, Santa Cruz, CA, USA), anti-complex IV (MitoSciences, Eugene, OR, USA), anti-AMPK α2 (Upstate, Lake Placid, NY, USA) and polyclonal anti-β-actin (Cell signaling, Pickering, ON, Canada) diluted 1/1000. Polyclonal anti-phospho-AMPK (Cell signaling, Pickering, ON, Canada) was diluted at 1/500. The secondary antibodies were anti-mouse and anti-rabbit antibodies coupled to horseradish peroxidase, respectively. Proteins were visualized using an enhanced luminescent reagent and exposed to autoradiograph film. β-actin and Troponin T were used as a loading control and a marker of myotube differentiation, respectively. Expression of proteins was quantified by density analysis using ImageJ Launcher Software.

### Cell redox environment

Cell redox environment following 7 days of differentiation in HG, LG or GAL was assessed using the MTT Assay Kit (Millipore, Billerica, MA, USA).

### ATP assay

ATP content in myotubes differentiated in HG, LG or GAL for 7 days was assessed using the StayBrite ATP assay kit (Biovision, Mountain View, CA, USA).

### HPLC Determination of Cellular Lactate Release

Myoblasts were seeded at 200,000 cells per well of a 6-well plate. Upon reaching ∼90% confluency, cells were differentiated in their respective differentiation medium (HG, LG or GAL) for 7 days. On the 6^th^ day post differentiation the medium was changed and then collected 24 h later for analysis. For experiments, 1 ml of media was treated with 50 µl of 0.5% (v/v) perchloric acid solution. Samples were then centrifuged at 12,000 g for 10 min and subsequently filtered with a sterile syringe and 0.2 µm filtration unit. Samples were injected into an Agilent 1100 Series HPLC system equipped with a C_18_ reverse phase hydrophilic column (Phenomenex) operating at a flow rate of 0.7 ml/min. Lactate was detected using an Agilent variable wavelength detector set to 210 nm. The retention time of lactate was confirmed by injection standard lactate solutions. Lactate levels were quantified by injection varying concentrations of standard lactate solutions and the use of Agilent ChemStation software.

### Extracellular Flux (XF) Analysis of Cellular Metabolic Characteristics

20,000 cells per well were plated in a XF24-well plate (Seahorse Bioscience, North Billerica, MA, USA), grown to ∼90% confluency and differentiated for 7 days in HG, LG or GAL. Myotubes were then incubated for 45 min at 37°C at ambient CO_2_ in HCO_3_-free DMEM (pH 7.4) containing 4 mM glutamine, 1 mM pyruvate and 25 mM glucose (HG), or 5 mM glucose (LG) or 10 mM galactose (GAL). Oxygen consumption rates (OCR) were determined *in situ* using a Seahorse Extracellular Flux Analyzer. Baseline oxygen consumption rate was measured 3 times for 4 min each separated by a 2 min wait and a 2 min mix. Following the measurement of basal respiration, oligomycin (600 ng/ml) (Sigma–Aldrich, Oakville, ON, Canada) was injected into each well, followed by 3 cycles of: 2 min mix, 2 min wait and 4 min measurement to measure state 4 (non-phosphorylating) respiration. Then, FCCP (1 µM) (Sigma–Aldrich, Oakville, ON, Canada) was injected into each well, followed by 3 cycles of: 2 min mix, 2 min wait and 2.5 min measurement to measure maximal respiration. Finally, antimycin A (4 µM) (Sigma–Aldrich, Oakville, ON, Canada) was injected into each well, followed by 3 cycles of: 2 min mix, 2 min wait and 3 min measurement to measure extramitochondrial O_2_ consumption. The cells were then collected for determinations of protein content (Bradford assay). The data are presented as: mitochondrial OCR (basal, oligomycin or FCCP OCR are reported following the subtraction of OCR in the presence of antimycin) and expressed per µg of total cellular protein.

### Mitochondrial yield, Citrate Synthase (CS) and COX Activity Assays

Myoblasts were grown in a 75-cm^2^ flask to ∼90% confluency and then differentiated in their respective differentiation media (HG, LG or GAL) for 7 days. Intact mitochondria were isolated using a kit (MITOISO; Sigma–Aldrich, Oakville, ON, Canada). Mitochondrial and total cellular protein levels were determined by a BCA (bicinchoninic acid) assay. Mitochondrial yield was expressed as µg of mitochondrial protein per µg of total cellular protein. CS activity was assayed in lysed mitochondria using a kit (CSO720; Sigma–Aldrich, Oakville, ON, Canada) with 0.5 mmol/l oxaloacetate, 0.3 mmol/l acetyl-CoA, 0.1 mmol/l of 5, 5′-dithiobis 2-nitro-benzoic acid. Enzyme activity was monitored by recording the changes in absorbance at 412 nm over 1.5 min. COX activity was measured in intact mitochondria using a kit (Cytocox1; Sigma–Aldrich, Oakville, ON, Canada) based on the decrease in absorbance over 3 min at 550 nm of ferrocytochrome C caused by its oxidation to ferricytochrome C by COX. Enzyme activities were normalized to protein content.

### Cardiolipin staining and quantification

10N-nonyl acridine orange (NAO, Sigma–Aldrich, Oakville, ON, Canada) staines the mitochondrial phospholipid cardiolipin [44]. NAO is used as a mitochondrial content marker since it is essentially independent of the mitochondrial membrane potential [45]. After differentiation in HG, LG and GAL for 7 days, myotubes were fixed with paraformaldehyde at 4% in PBS for 10 min, and then cardiolipin was stained by NAO (1 µM) for 30 min, in the dark, at room temperature. The dye was then washed 3 times with PBS, and mitochondrial content quantified in a fluorometer (emission: 535 nm, excitation: 485 nm).

### Statistical analyses

Data are presented as mean ±SEM. Statistical analyses were performed using Statview 5.0 (SAS institute Inc, Cary, NC, USA). One-way or 2-ways ANOVAs with Fisher's protected least significant difference post-hoc test were used to assess statistical differences. P<0.05 was considered to be significant.
