# Correction: Galactose Enhances Oxidative Metabolism and Reveals Mitochondrial Dysfunction in Human Primary Muscle Cells

**DOI:** 10.1371/annotation/4a3c143c-7338-4ed3-8fc4-a21526fd05a6

**Published:** 2012-01-26

**Authors:** Céline Aguer, Daniela Gambarotta, Ryan J. Mailloux, Cynthia Moffat, Robert Dent, Ruth McPherson, Mary-Ellen Harper

There was an error in the oxygen consumption rate (OCR) unit of measurement. The correct unit of measurement is pmol. 

