# Correction: Galactose Enhances Oxidative Metabolism and Reveals Mitochondrial Dysfunction in Human Primary Muscle Cells

**DOI:** 10.1371/annotation/eb51f7a7-a8fd-45a3-9df0-e6080c47fe06

**Published:** 2012-01-26

**Authors:** Céline Aguer, Daniela Gambarotta, Ryan J. Mailloux, Cynthia Moffat, Robert Dent, Ruth McPherson, Mary-Ellen Harper

There was an error in the oxygen consumption rate (OCR) unit of measurement. The correct unit of measurement is pmol.

